# Spinal cholinergic interneurons differentially control motoneuron excitability and alter the locomotor network operational range

**DOI:** 10.1038/s41598-018-20493-z

**Published:** 2018-01-31

**Authors:** Maria Bertuzzi, Konstantinos Ampatzis

**Affiliations:** Department of Neuroscience, Karolinska Institutet, 171 77 Stockholm, Sweden

## Abstract

While cholinergic neuromodulation is important for locomotor circuit operation, the specific neuronal mechanisms that acetylcholine employs to regulate and fine-tune the speed of locomotion are largely unknown. Here, we show that cholinergic interneurons are present in the zebrafish spinal cord and differentially control the excitability of distinct classes of motoneurons (slow, intermediate and fast) in a muscarinic dependent manner. Moreover, we reveal that m2-type muscarinic acetylcholine receptors (mAChRs) are present in fast and intermediate motoneurons, but not in the slow motoneurons, and that their activation decreases neuronal firing. We also reveal a strong correlation between the muscarinic receptor configuration on motoneurons and the ability of the animals to locomote at different speeds, which might serve as a plasticity mechanism to alter the operational range of the locomotor networks. These unexpected findings provide new insights into the functional flexibility of motoneurons and how they execute locomotion at different speeds.

## Introduction

Neural networks in the spinal cord are responsible for the generation and execution of movements^1–4^. These spinal cord networks in zebrafish are organized in distinct functional microcircuit modules^5,6^ with defined operational ranges, whose sequential activation increases the speed of locomotion^5^. The activity of the spinal neuronal circuits is regulated by a range of neuromodulatory systems^7^ to adjust the final motor output. One such prominent neuromodulatory system is the cholinergic system^8,9^. In rodents, cholinergic neuromodulation increases motoneuron excitability^8,10^ in a task-dependent manner^9^, which is exclusively mediated by cholinergic interneurons (V0c)^9^. Whether cholinergic interneurons are present in the zebrafish spinal cord, and how they regulate the activity of the distinct motoneuron pools (slow, intermediate and fast) during locomotion is, however, unknown.

In the vertebrate spinal cord, motoneuron and interneuron cholinergic transmission has been implicated in modulation of neuronal excitability, through the activation of two distinct types of receptors, the muscarinic and nicotinic acetylcholine receptors^11,12^. In mammals, acetylcholine release increases motoneuron excitability through the activation of metabotropic muscarinic acetylcholine receptors (mAChRs)^8,13–15^. Although m2 type muscarinic acetylcholine receptor (m2-mAChR) activation is reported to mediate mammalian motoneuron hyperexcitability^8–10^, numerous studies also demonstrate m2-mAChR-mediated inhibitory actions, in several neuronal populations^16–18^, including motoneurons^19^. Moreover, m2-mAChRs are found to be predominantly expressed in large motoneurons^20,21^. This suggests that only a subset of motoneurons are sensitive to cholinergic modulation via the m2-mAChRs. In order to resolve the question of whether m2 receptors are present in all motoneuron pools (slow, intermediate and fast), and determine how activation of the m2-mAChRs influences the motoneuron functionality, we used the accessible neuro-muscular configuration of the adult zebrafish^22^.

Using a combination of anatomical, electrophysiological, pharmacological, *ex-vivo* and *in-vivo* behavioral approaches in the adult zebrafish, we reveal the existence of a population of spinal cord interneurons, the cholinergic interneurons. Furthermore, we observe a strong correlation between the muscarinic receptor configuration on motoneurons and the ability of the animals to locomote at different speeds, involving the activation of distinct mAChR subtypes, suggesting that this might be a plasticity mechanism to change the operational ranges of the locomotor networks.

## Results

### Zebrafish spinal cholinergic system organization

**Figure 1:**
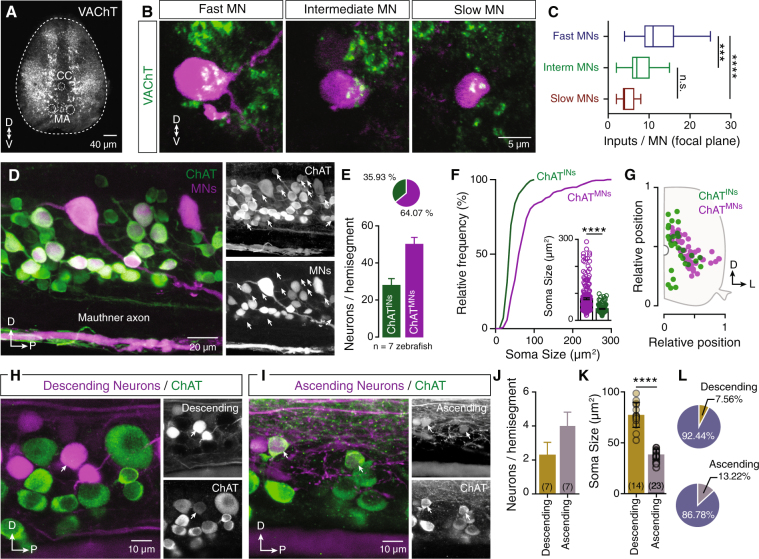
Organization of spinal cholinergic system. (**A**) Distribution of cholinergic terminals within the adult zebrafish spinal cord. (**B**) Single optical sections show the cholinergic synapses onto different types of motoneurons (MNs). (**C**) Cholinergic (ACh) input is different between motoneuron (MN) classes, (n_slow_ = 10, n_interm_ = 27, n_fast_ = 30 neurons; n = 5 zebrafish). Quantification of inputs on each MN obtained from a single focal plane images. (**D**) Whole mount spinal cord immunohistochemistry reveals that a fraction of ChAT^+^ neurons are not backfilled motoneurons. Arrows indicate ChAT^+^MN^−^ neurons (Cholinergic interneurons). (**E**) Quantification of the adult zebrafish cholinergic neurons, per spinal cord hemisegment, that are motoneurons or interneurons (n = 7 zebrafish). (**F**) Cholinergic interneurons (ChAT^INs^) have smaller soma sizes compared to motoneurons (ChAT^MNs^). (**G**) Representative example of the distribution pattern of cholinergic interneurons (ChAT^INs^) and motoneurons (ChAT^MNs^) in the adult zebrafish spinal cord. (**H**) Injection of a retrograde dextran tracer in spinal segment 18 reveals the descending interneurons located in spinal cord segment 15. Few descending neurons that are cholinergic (ChAT^+^) were found in the spinal hemisegment of adult zebrafish. Arrow indicates cholinergic descending interneuron. (**I**) Injection of dextran retrograde tracer in spinal segment 12 reveals the ascending interneurons in spinal cord segment 15. Arrows indicate a small number of retrograde traced neurons that are cholinergic interneurons. (**J**) Analysis of the number of the cholinergic interneurons that possess a descending (2.28 ± 0.28 neurons/hemisegment, n = 7 zebrafish) or an ascending (4 ± 0.3 neurons/hemisegment, n = 7 zebrafish) axon. (**K**) The descending and ascending cholinergic interneurons have non-overlapping soma sizes (t = 13.4, *p* < 0.0001, n = 37 neurons), suggesting that different populations of cholinergic interneurons are ascending or descending in adult zebrafish spinal cord. (**L**) Percentage of the descending and ascending cholinergic interneurons per hemisegment of spinal cord. CC, central canal; D, dorsal; L, lateral; MA, Mauthner axon; P, posterior; V, ventral. Data are presented as mean ± SD; ***p < 0.001; ****p < 0.0001; n.s., non-significant.

We first examined the distribution pattern of cholinergic terminals (VAChT^+^) in the adult zebrafish spinal cord. Multiple cholinergic terminals were detected in the dorsal horn, neuropil and motor column (Fig. 1A). Using the accessible neuro-muscular configuration of the adult zebrafish, we dissected distinct functional motoneuron pools^22^. All retrogradely traced secondary motoneuron types (slow, intermediate and fast) received abundant cholinergic innervation (Fig. 1B). To determine whether this innervation differs among functionally different pools of secondary motoneurons, we analyzed the number of cholinergic terminals (VAChT^+^) in close proximity to motoneuron cell bodies (see Methods). Each fast motoneuron receives a significantly higher number of putative cholinergic inputs than each intermediate and slow motoneurons (*One-way* ANOVA, F_2,64_ = 16.69, *p* < 0.0001, n = 67 neurons; Fig. 1C). Furthermore, our analysis showed that these cholinergic synapses on motoneurons do not display the typical morphology of the mammalian “c-boutons”^9,10,23,24^. To investigate the origin of this input, we retrogradely labeled the brain neurons descending to spinal cord (fluorescent dextran tracer; see Methods) and subsequently immunolabeled cholinergic neurons (Choline Acetyltransferase (ChAT) immunoreactivity; n = 8 brains). Our analysis revealed that none of these neurons were cholinergic as shown by the co-localization experiments (Fig. S1).

Having established that spinal cord cholinergic innervation is not provided by brain descending neurons, we hypothesized that spinal cord cholinergic interneurons account for the cholinergic input to motoneurons. This hypothesis is supported by the morphology of the adult zebrafish motoneurons, which lack axonal collaterals^22^, that are recurrent axons that branch off from the main axon. In mammals, motoneuron collaterals form synaptic contacts with the Renshaw cells and other motoneurons^11,25^. Renshaw cells have not been reported in the zebrafish spinal circuits, and zebrafish motoneuron-motoneuron communication is meditated through dendro-dendritic electrical gap junctions^26^. To test our hypothesis, we injected a retrograde tracer into zebrafish spinal cord ventral roots and combined with ChAT immunoreactivity (Fig. 1D). Given that all zebrafish motoneuron axons exiting from the same ventral root correspond to the spinal hemisegment, this approach enabled us to reveal the spinal motoneurons (axial and fin; Fig. 1D). We identified a significant fraction (35.93 ± 1.6%; Fig. 1E), of small to medium size non-motoneuron cholinergic neurons (ChAT^INs^, 42.47 ± 1.4 μm^2^; Fig. 1F), which were distributed throughout the motor column (Fig. 1G). Although the ChAT^INs^ were distributed throughout the motor column, ~80% were found in the dorsal part of the motor column close to the central canal (Fig. 1G).

To assess whether spinal cholinergic interneurons innervate targets located in rostral or caudal spinal cord segments, we identified cholinergic interneurons (segment 15) as ascending or descending, after injection of a retrograde tracer (dextran) into segments 12 or 18, respectively (n = 14 zebrafish; Fig. 1H,I). 24 h after the tracer injection we processed the tissue for ChAT immunostaining. We observed that 7.5% of ChAT^INs^ were labelled as descending neurons (Fig. 1J,L), and 13.2% of ChAT^INs^ were determined as ascending neurons (Fig. 1J,L), indicating that 20.7% of the cholinergic interneurons project to other spinal cord segments. The significant difference between the soma size of the ascending and descending cholinergic interneurons suggest that they form two non-overlapping neuronal subpopulations (Fig. 1K). Overall, our data suggest that zebrafish spinal motoneurons receive unevenly distributed cholinergic input from a local spinal source, represented by cholinergic interneurons.

### Muscarinic receptors differentially alter motoneuron excitability

**Figure 2:**
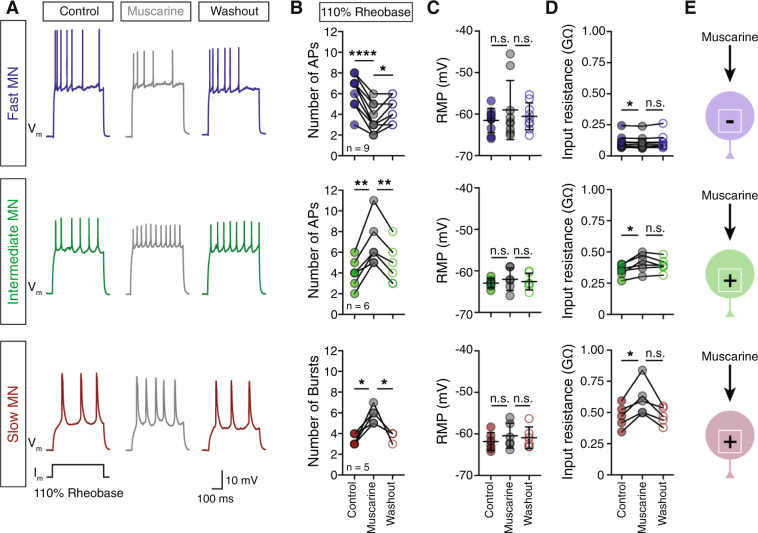
Muscarinic receptors differentially alter motoneuron excitability. (**A**) Representative traces of motoneuron (MN) firing in responses to 110% of rheobase somatic current injection, before (colored traces) after bath application of muscarine (15 μM; gray traces) and followed by washout (colored traces). (**B**) In the presence of muscarine the fast motoneurons significantly reduce the number of action potentials (APs) in response to 110% of rheobase current injection, while both the intermediate and the slow motoneurons increase their firing rate. During washout, the motoneurons partially recover (n_fast_ = 9 neurons, n_intermediate_ = 6 neurons, n_slow_ = 5 neurons). (**C**) During the application of muscarine (gray circles) and after washout (open colored circles) there is no significant change in the resting membrane potential of the motoneurons. (**D**) Bath application of muscarine alters the input resistance of all motoneuron types. The input resistance was decreased in the fast motoneurons and increased in the intermediate and slow motoneurons. (**E**) Overview of the change in excitability of different types of MNs in response to muscarine. The sign represents the alteration of the MN excitability (+ = increase, − = decrease). Data are presented as mean ± SD; *p < 0.05; **p < 0.01; ****p < 0.0001; n.s., non-significant.

In mammals, acetylcholine increases the excitability of spinal motoneurons through the activation of muscarinic receptors^8^. To determine whether zebrafish motoneuron excitability is responsive to activation of mAChRs we obtained whole-cell current-clamp recordings from different pools (slow, intermediate and fast) of axial secondary motoneurons. Since the fast and intermediate motoneurons discharge single action potentials (APs), whereas slow motoneurons fire in bursts of APs^22,27^, the excitability of motoneurons was assessed by examining their response (number of single APs or number of bursts) to steps of supra-threshold depolarizing current pulses (500 ms, increments of 10% from rheobase), before and after the application of muscarine, a non-selective mAChR agonist. Muscarine (15 μM) significantly increased the firing rate of both slow (*one-way* ANOVA repeated measures, F_1.319,5.277_ = 16, *p* = 0.021, n = 5 out of 5) and intermediate (*one-way* ANOVA repeated measures, F_1.269,6.344_ = 24.36, *p* = 0.0079, n = 6 out of 6 neurons) motoneurons (Fig. 2A,B,E). This was accompanied by an increase in the input resistance (slow MNs: *one-way* ANOVA repeated measures, F_1.059,4.237_ = 13.34, *p* = 0.0227; intermediate MNs: *one-way* ANOVA repeated measures, F_1.493,7.466_ = 12.64, *p* = 0.0258; Fig. 2D), without significantly altering the resting membrane potential (Fig. 2C). In contrast, the fast motoneurons showed a decrease in excitability, resulting in fewer action potentials (*one-way* ANOVA repeated measures, F_1.986,15.89_ = 38.05, *p* < 0.0001, n = 9 out of 9 neurons; Fig. 2A,B,E) associated with a significant decrease of the input resistance (*one-way* ANOVA repeated measures, F_1.12,8.958_ = 4.46, *p* = 0.0141, Fig. 2D). Finally, the observed changes in electrical properties of the motoneurons are consistent with previously reported alterations of mammalian motoneurons in response to muscarine^15^ (Fig. S2A–F).

### Motoneurons possess different muscarinic receptor subtypes

**Figure 3:**
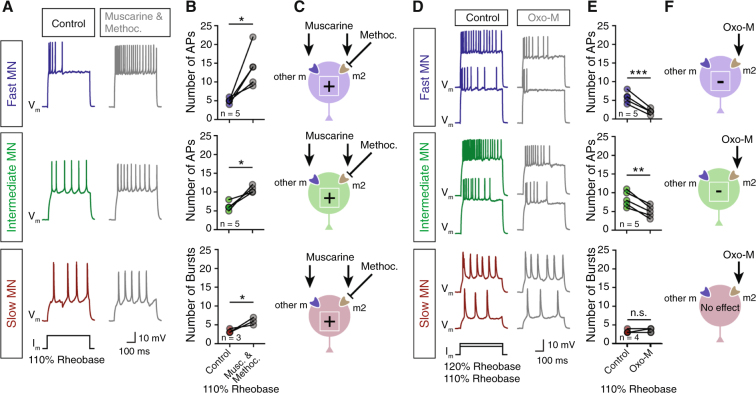
Motoneurons possess different muscarinic receptor subtypes. (**A**) Traces of motoneuron (MN) firing in response to 110% of rheobase current injection, before (colored traces) and after bath application of a mixture of muscarine (15 μM) and methoctramine (10 μM; gray traces). (**B**,**C**) Co-application of muscarine and methoctramine produce hyperexcitability in all MN pools (n_fast_ = 5 neurons, n_intermediate_ = 5 neurons, n_slow_ = 3 neurons), derived from the antagonistic effect on m2-mAChRs. (**D**) Representative traces of motoneuron (MN) firing in response to 110% and 120% of rheobase current injections, before (colored traces) and after bath application of oxotremorine-M (Oxo-M; 20 μΜ; gray traces) which preferentially activates the m2-mAChRs. (**E**,**F**) In the presence of oxotremorine-M, excitability decreases in the fast and intermediate motoneurons, whereas the slow motoneurons do not change their firing rate in response to 110% of rheobase current injection (n_fast_ = 5 neurons, n_intermediate_ = 5 neurons, n_slow_ = 4 neurons). *p < 0.05; **p < 0.01; ***p < 0.001; ****p < 0.0001; n.s., non-significant.

We speculated that the differential motoneuron excitability we observed might arise from the presence of different mAChR subtypes in the motoneurons. To test if m2-mAChRs alone mediate the changes in motoneuron excitability, we applied a mixture of muscarine (15 μM) and the selective m2-mAChR antagonist methoctramine^28^ (10 μM). We recorded an increase in excitability in all motoneuron pools, fast (paired *t-*test, t = 3.64, *p* = 0.0219, n = 5 out of 5 neurons; Fig. 3A–C), intermediate (paired *t-*test, t = 4.32, *p* = 0.0228, n = 4 out of 4 neurons) and slow (paired *t-*test, t = 4.00, *p* = 0.0161, n = 3 out of 3 neurons; Fig. 3A–C), suggesting that the activation of all muscarinic receptors, except for m2-mAChR, induces this opposite effect observed in fast motoneurons. From these experiments, we hypothesize that the activation of m2-mAChRs decreases the excitability of motoneurons. To further assess whether m2-mAChRs are present in all classes of motoneurons, and to validate their contribution in modulating motoneuron excitability, we applied oxotremorine-M (Oxo-M; 20 μΜ), an m2-mAChR preferential agonist^29^. In response, the fast (paired *t-*test, t = 10.00, *p* = 0.0005, n = 5 out of 5 neurons) and intermediate (paired *t-*test, t = 6.66, *p* = 0.0026, n = 5 out of 5 neurons) motoneurons exhibited reduced firing (Fig. 3D–F), whereas, slow motoneurons were unaffected (paired *t-*test, t = 1.00, *p* = 0.391, n = 4 out of 4 neurons; Fig. 3D–F), suggesting that they do not express the m2-mAChR subtype. Moreover, we additionally confirmed the presence of m2-mAChRs in motoneurons anatomically. Immunoreactivity for the m2-mAChRs was strong in fast motoneurons and modest in intermediate motoneurons (*One-way* ANOVA, F_2,35_ = 51.32, *p* < 0.0001, n = 38 neurons; Fig. S3A,B). In contrast, slow motoneurons were weakly labeled for the m2-mAChR, supporting previous reports^20^ (Fig. S3A,B). These results indicate that multiple mAChR subtypes are expressed in a motoneuron type-dependent manner.

### Activation of motoneuron muscarinic receptors controls fast locomotion

**Figure 4:**
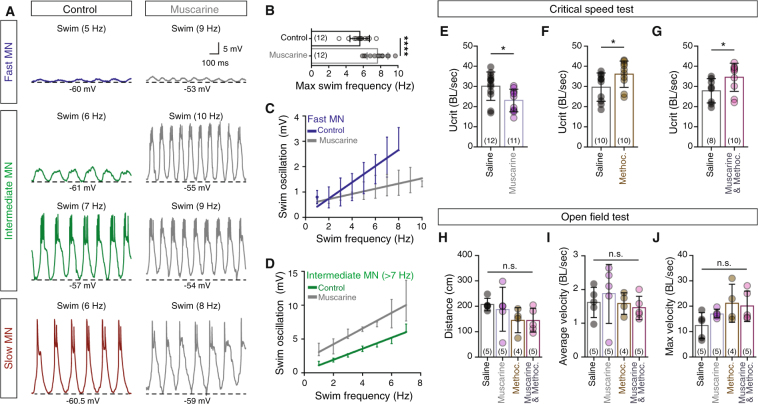
Activation of motoneuron muscarinic receptors controls fast locomotion. (**A**,**B**) During fictive locomotion, motoneurons display membrane potential oscillations. The highest swimming frequency increases in the presence of muscarine (15 μM; gray traces) during fictive locomotion (*ex-vivo* experiments). Dashed line indicates the membrane potential of the lower part of the oscillations. (**C**) Averaged slopes of the membrane potential oscillations in relation to different swimming frequencies of fast motoneurons in the absence (blue line), or presence (gray line) of muscarine. The data are presented as interpolation curve (middle line ± SD). (**D**) Slopes showing the increasing amplitude of the membrane potential oscillations of the intermediate motoneurons recruited above 7 Hz, in relation to the swimming frequency, before and after the application of muscarine. After muscarine (gray line) the amplitude of the membrane oscillations was significant increased (F = 856.6, *p* < 0.0001), in comparison to the control (green line). The data are presented as interpolation curve (middle line ± SD). (**E**–**G**) Intraperitoneal administration of muscarine reduces the U_crit_ (BL = 1.84 ± 0.11 cm; n = 23 zebrafish), while methoctramine increased the maximum swimming speed (BL = 1.68 ± 0.1 cm; n = 20 zebrafish). Co-administration of muscarine and methoctramine increased the maximum obtained swimming speed (BL = 1.71 ± 0.12 cm; n = 18 zebrafish). (**H**–**J**) *In-vivo* monitoring of adult zebrafish locomotor behavior. Muscarine and/or methoctramine administration was not found to affect the distance traveled, the average velocity and the maximum velocity of the studied animals (n = 19 zebrafish). The data were normalized to body lengths (BL)/sec. Data are presented as mean ± SD; *p < 0.05; ****p < 0.0001; n.s., non-significant.

Given that motoneuron excitability can retrogradely influence the upstream locomotor network function (presynaptic release, generation of action potentials, swimming duration and frequency) through gap junctions^26^, we tested the functional significance of motoneuron mAChR activation during locomotion. For this, we used the adult zebrafish *ex-vivo* preparation^5,22,26,27^, where we can induce locomotor activity at different swimming speeds by electrical stimulation of the brain descending axons. Bath application of muscarine facilitated fictive locomotor activity and increased the highest reached swimming frequency by 26.8 ± 5.8% (paired *t-*test, t = 6.67, *p* < 0.0001, n = 12; Fig. 4A,B). It should be noted that the amplitude of the swimming membrane potential oscillations during locomotion is related to swimming frequency^27^. In the presence of muscarine, the slope representing the correlation between oscillation amplitude and frequency was decreased in the fast motoneurons (Fig. 4C) and enhanced in the intermediate motoneurons that were recruited above 7 Hz (Fig. 4D). Overall, our findings demonstrate that activation of spinal muscarinic receptors facilitates the recruitment of the intermediate motoneuron-interneuron module while hindering the recruitment of the fast locomotor module.

Finally, we sought to understand the *in-vivo* behavioral functions of the activation of mAChRs. Therefore, we subjected zebrafish to a critical speed test (see Methods; Fig. S4B). Critical speed (U_crit_) is a measure of the highest sustainable swimming speed that a fish can reach^30^. Intraperitoneal administration of muscarine (50 μM, 525 ng/g BW; Fig. S4A) significantly reduced the highest sustainable swimming speed (unpaired *t-*test, t = 2.65, *p* = 0.014, n = 23 zebrafish; Fig. 4E). In contrast, methoctramine treated animals (40 μM, 2.2 mg/g BW) increased the highest locomotor speed (unpaired *t-*test, t = 2.12, *p* = 0.047, n = 20 zebrafish; Fig. 4F). Similarly, exogenous activation of all muscarinic receptors except the m2-mAChRs, achieved by co-administration of muscarine (50 μM) and methoctramine (40 μM), increased the maximum locomotor speed obtained (unpaired *t-*test, t = 2.15, *p* = 0.046, n = 18 zebrafish; Fig. 4G).

To test the effect of our pharmacological treatments on locomotion under normal conditions, animals were subjected to an open field test (see Methods; Fig. S4C). We observed that similar treatment did not affect the regular locomotor behavior (distance traveled, average velocity and maximum velocity; Fig. 4H–J), suggesting that the differences in critical speed observed here are due to the effect on the spinal locomotor circuit. These data show that systemic stimulation of m2-mAChRs causes a reduction in the maximum swimming speed to 76.5 ± 6.7% of the of the U_crit_, corresponding to the zebrafish optimum speed^31^, where swimming relies only on the activity of the slow and intermediate neuro-muscular system, and not on the fast. Our analysis cannot rule out the possibility of an additional direct effect of mAChR activation in the premotor neurons, however, as any alteration in motoneuron excitability will retrogradely affect the excitability and pre-synaptic release of glutamate from V2a interneurons^26^, which will influence premotor network functionality. Taken together, our results suggest that cholinergic modulation acts on locomotor network to modify the speed of locomotion mainly through possibly the engagement of the fast motoneuron-interneuron module.

## Discussion

**Figure 5:**
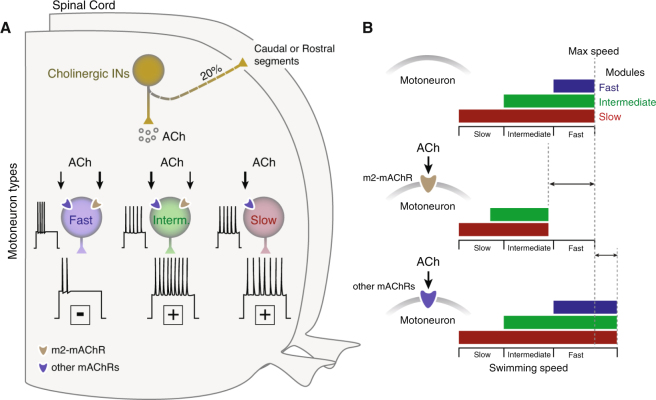
Differential organization of cholinergic input to MNs permits changes of the operational range of locomotor circuit. (**A**) Acetylcholine release from spinal cholinergic interneurons affects the excitability of different types of motoneurons in a mAChR dependent manner. Our results suggest that acetylcholine can increase the slow motoneuron pool excitability since these neurons do not express the m2-mAChRs. In contrast, the intermediate and fast motoneurons express different subtypes of mAChRs that differentially alter their excitability. (**B**) During locomotion, the different pools of motoneurons are recruited in a stepwise manner from slow, to intermediate, to fast to cover the full range of swimming frequencies. The activation of different mAChRs can allow the animals to adjust the recruitment of the locomotor microcircuit modules and alter the operational range of these networks.

Our findings suggest that motoneurons receive cholinergic input exclusively from spinal interneurons. Acetylcholine shifts motoneuron excitability through the parallel activation of different mAChRs subtypes (Fig. 5A). Moreover, activation of the muscarinic cholinergic pathway selectively alters the operational range of locomotion (Fig. 5B). Overall, this work provides novel insights into how intraspinal acetylcholine release can modify via muscarinic receptors the functionality of the spinal circuitry, which requires synaptic specificity and temporal precision to generate locomotion at different speeds^5,22^.

One of our major findings is establishing the existence of cholinergic interneurons in the zebrafish spinal cord and identifying them as the exclusive source of the cholinergic spinal input. Earlier studies showed the existence of cholinergic interneurons in several vertebrate species^8,9,32–35^. Moreover, the zebrafish cholinergic system is well characterized^36,37^, however the presence of zebrafish spinal cord cholinergic interneurons has not been previously reported. While a number of cholinergic neurons that do not express the motoneuron identity marker *Islet1* were identified earlier^38^, it has been proposed that these small cholinergic neurons correspond to motoneurons, other than the ones that innervate the axial muscles^37^. Our analysis cannot rule out the possibility that cholinergic interneurons can form several different subpopulations in zebrafish spinal cord. In the mammalian spinal cord, motoneuron cholinergic neuromodulation is mediated by the V0c interneurons^9^. However, previous attempts to reveal the existence of V0c interneurons in zebrafish spinal cord were ineffective^39^. Since V0c interneurons have been shown to be the exclusive source of cholinergic c-bouton input to mammalian motoneurons^9^, our data implicate the activity of spinal cholinergic interneurons in the cholinergic modulation of motoneurons also in the zebrafish.

The overall activation of muscarinic receptors has been shown to produce slow EPSPs to increase motoneuron excitability^8,13–15,40,41^ via activation of the m2-mAChR subtype^8^. On the other hand, numerous studies have shown that activity of the m2-mAChRs is associated with inhibitory actions in the nervous system^16–19^ since they elicit muscarinic dependent IPSPs in the mammalian striatum^42^. Our findings challenge previous conclusions that activation of motoneuron m2-mAChR is primarily required to increase motoneuron excitability. By pharmacologically manipulating the m2-mAChRs in different motoneuron pools, we found that m2-mAChR activation reduces motoneuron excitability and other receptor subtypes can account for the observed rise in motoneuron firing. In line with this idea, various subtypes of mAChRs have been characterized in spinal cord neurons^18,43–45^ and it is also well documented that many nerve cells contain more than one subtype of muscarinic receptor^45–47^. The exact muscarinic receptor subtype responsible for the elevated excitability of adult zebrafish motoneurons remains to be identified. However, it has been suggested that the excitatory effects of muscarinic agonists on neonatal rat motoneurons are mediated through the m3-mAChRs^19^. In support of our observations, Jordan *et al*.^45^ showed that m2-mAChRs and m3-mAChRs are both involved in the cholinergic modulation of locomotion in mammals. They showed that application of the m2-mAChR antagonist methoctramine, increased the locomotor rhythm, whereas application of m3-mAChR antagonist reduced, and finally blocked, the locomotor activity^45^. Our results show that different muscarinic receptor subtypes in the membrane of motoneurons can play a unique role in the regulation of neural activity involved in the control of locomotion.

A general question about the cholinergic control of locomotion is made addressable in our work: how does the muscarinic acetylcholine receptor configuration gate the execution of locomotor behavior at different speeds? It has been proposed that the cholinergic modulation of motoneurons is activity dependent^9^. In favor of this idea, our work revealed that pharmacological manipulations of muscarinic receptors *ex-vivo* and *in-vivo* only affect the locomotor performance of zebrafish at higher swimming speeds, which are primarily associated with the engagement and function of the fast neuromuscular system. Thus, the mechanisms we identify here suggest an additional level of control during high-energy-demand locomotion, such as fast swimming.

Given the emerging evidence regarding the importance and functional repertoire of spinal cholinergic input in the control of motor behaviors, it is not surprising that cholinergic synapses have been implicated in spinal cord injury^24,45,48^ and motor disease^23,49,50^. However, no causal link has yet been found between alterations in the number and size of cholinergic synapses, acetylcholine release and muscarinic receptor activation. Therefore, uncovering the mechanisms by which acetylcholine modifies the activity of spinal neurons is of significant biological and medical interest. We find that methoctramine treated animals are able to operate at higher swimming speeds and, in addition, activation of m2-mAChRs reduces the excitability of the motoneurons, and potentially of other spinal cord neurons, and their ability to produce the appropriate muscle force. Application of methoctramine is found to increase the locomotor frequency also in mammals^45^. Moreover, application of cholinergic receptor antagonists following spinal cord injury was found to advance locomotor activity, suggesting that adaptive alterations of the spinal cholinergic system obstruct the generation and execution of locomotion^45^. In a mouse model of amyotrophic lateral sclerosis (ALS), methoctramine treatment counteracts the loss of muscle force during the pre-symptomatic stages of the disease^50^, and is likely to be mediated through the blocking of m2-mAChRs, increasing the excitability of spinal neurons participating in the generation of movement. In conclusion, our results provide a novel contribution to existing knowledge, and further understanding of a possible causal relationship between activation of muscarinic receptors and locomotion, highlighting their potential as targets for innovative therapeutic strategies.

## Methods

### Animals

All animals were raised and kept in a core facility at the Karolinska Institute according to established procedures. Adult zebrafish (*Danio rerio*; 8–10 weeks old; length, 17–19 mm; weight, 0.025–0.045 g) wild type (AB/Tübingen) was used in this study. All experimental protocols were approved by the local Animal Research Ethical Committee, Stockholm and were performed in accordance with EU guidelines.

### Motoneuron and descending/ascending neuron labeling

Zebrafish (n = 30) of either sex were anaesthetized in 0.03% tricaine methane sulfonate (MS-222, Sigma-Aldrich). Retrograde labeling of axial motoneurons was performed using dye injections with tetramethylrhodamine-dextran (3000 MW; ThermoFisher, D3307) in specific muscle fiber types (slow, intermediate or fast). In addition, retrograde labeling of motoneurons was performed using similar procedure to spinal cord ventral roots. To label the neurons descending from the brain to the spinal cord the tracer was injected using dye-soaked pins in the spinal cord at approximately the level of the 6–8th vertebra. Finally, for the investigation of descending and ascending cholinergic interneurons the tracer was injected in the spinal segment 18 (n = 7 zebrafish) or 12 (n = 7 zebrafish) respectively. Afterwards all the animals were kept for at least 24 h to allow the retrograde transport of the tracer. We evaluated the number of ChAT^+^ interneurons that were positive to the tracer on segment 15 of the adult zebrafish spinal cord.

### Immunohistochemistry

All animals were deeply anesthetized with 0.1% MS-222. We then dissected the spinal cords and/or the brains and fixed them in 4% paraformaldehyde (PFA) in phosphate buffer saline (PBS) (0.01 M; pH = 7.4) at 4 °C for 2–14 h. We performed immunolabelings in both whole mount and cryosections. For cryosections, the tissues were removed carefully and cryoprotected overnight in 30% (w/v) sucrose in PBS at 4 °C, embedded in OCT Cryomount (Histolab), rapidly frozen in dry-ice-cooled isopentane (2-methylbutane; Sigma) at approximately −35 °C, and stored at −80 °C until use. Transverse coronal plane cryosections (thickness 25 μm) of the tissue were collected and processed for immunohistochemistry. The tissue was washed three times for 5 min in PBS. Nonspecific protein binding sites were blocked with 4% normal donkey serum with 1% bovine serum albumin (BSA; Sigma) and 0.5% Triton X-100 (Sigma) in PBS for 30 min at room temperature (RT). Primary antibodies were diluted in 1% of blocking solution and applied for 24–90 h at 4 °C. For primary antibodies, we used goat anti-ChAT (1:200; Millipore, AB144P, RRID: AB_2079751), guinea pig anti-VAChT (1:1000; Millipore, AB1588, RRID: AB_11214110), and rabbit anti-m2 mAChRs (1:700; Alomone, AMR-002, RRID: AB_2039995). After thorough buffer rinses the tissues were then incubated with the appropriate secondary antibodies diluted 1:500 in 0.5% Triton X-100 (Sigma) in PBS overnight at 4 °C. We used Alexa Fluor–conjugated secondary antibodies anti-goat 488 (ThermoFisher Scientific, A11055, RRID: AB_142672), anti-goat 568 (ThermoFisher Scientific, A11057, RRID: AB_142581), and anti-guinea pig 488 (ThermoFisher Scientific, A11073, RRID: AB_142018). Finally, the tissues were thoroughly rinsed in PBS and cover-slipped with fluorescent hard medium (VectorLabs; H-1400).

### Microscopy and image analysis

Imaging was carried out in a laser scanning confocal microscope (LSM 510 Meta, Zeiss). Cholinergic inputs on different motoneuron types were counted on single plan confocal images. Counting was performed in non-overlapping fields of spinal cord sections, in spinal cord segments 14–17. We defined the putative cholinergic inputs as number of large (>0.5 μm of diameter) VAChT-positive putatively cholinergic synapses, apposing the somata of motoneurons. The evaluation included 10 slow, 27 intermediate and 30 fast positive motoneurons collected from 4 adult zebrafish spinal cords. For the quantification of m2-mAChRs, all images were captured at identical exposure times in order to ensure the same illumination level. The intensity of m2-mAChRs immunoreactivity was evaluated in the obtained confocal pictures using the ImageJ image analysis software. A threshold of 0.18 of the normalized intensity was applied *post-hoc* to determine the tissue fluorescence background. The relative position of the somata of the neurons within spinal cord, was calculated in whole mount preparations, using the lateral, dorsal, and ventral edges of spinal cord as well as the central canal as landmarks. Analysis of all spinal cord neurons was performed between segments 14–17. The relative position was calculated using ImageJ. Examination of the descending neurons was performed from a series of coronal brain section, throughout the brain, without discarding any section from the analysis. The nomenclature used for the brain areas of descending neurons was based on the topological zebrafish brain atlas^51^. All figures and graphs were prepared with Adobe Photoshop and Adobe Illustrator (Adobe Systems Inc., San Jose, CA). All double-labeled images were converted to magenta-green immunofluoresence to make this work more accessible to the red-green color-blind readers.

### *Ex-vivo* preparation and electrophysiology

The dissection procedure has been described previously^5,22,26,27^. The preparations (n = 47) were then transferred to a recording chamber, placed lateral side up, and fixed with Vaseline. The chamber was continuously perfused with extracellular solution contained: 134 mM NaCl, 2.9 mM KCl, 2.1 mM CaCl_2_, 1.2 mM MgCl_2_, 10 mM HEPES, and 10 mM glucose, pH 7.8, adjusted with NaOH, and an osmolarity of 290 mOsm. All experiments were performed at an ambient temperature of 20–22 °C. For whole-cell intracellular recordings, electrodes (resistance, 9–13 MΩ) were pulled from borosilicate glass (outer diameter, 1.5 mm; inner diameter, 0.87 mm; Hilgenberg) on a vertical puller (PC-10 model, Narishige) and filled with intracellular solution containing the following: 120 mM K-gluconate, 5 mM KCl, 10 mM HEPES, 4 mM Mg_2_ATP, 0.3 mM Na_4_GTP, 10 mM Na-phosphocreatine, pH 7.4, adjusted with KOH, and osmolarity of 275 mOsm. Dextran-labeled MNs were visualized using a fluorescence microscope (Axioskop FS Plus, Zeiss) equipped with IR-differential interference contrast optics and a CCD camera with frame grabber (Hamamatsu) and were then targeted specifically. Intracellular patch-clamp electrodes were advanced in the exposed portion of the spinal cord through the meninges using a motorized micromanipulator (Luigs & Neumann) while applying constant positive pressure. Intracellular signals were amplified with a MultiClamp 700B intracellular amplifier (Molecular Devices) and low-pass filtered at 10 kHz. In current-clamp recordings, no bias current was injected. Only motoneurons that had stable membrane potentials at or below −48 mV fired action potentials to suprathreshold depolarizations and showed minimal changes in series resistance (<5%) were included in this study. Membrane potentials were corrected for a 6–9 mV liquid junction potential. The liquid junction potentials were calculated using the JPCalcW software (Molecular Devices). The following drugs were added to the physiological solution: non-selective muscarinic receptor agonist muscarine (15 μM; Sigma, M104), m2-type selective muscarinic receptor antagonist methoctramine (10 μM; Sigma, M105) and m2-type preferential muscarinic receptor agonist oxotremorine-M (20 μM; Sigma, O100). All drugs were dissolved as stock solutions in distilled water. For all the electrophysiological recordings data analysis was performed using Spike2 (version 7, Cambridge Electronic Design) or Clampfit (Molecular Devices) software.

### *In-vivo* swimming behavior

The swimming ability of zebrafish was tested using the open field test and the critical speed (U_crit_) test. U_crit_ is a measure of the highest sustainable swimming speed achievable by a fish. All zebrafish (n = 80) selected for the test displayed similar body length sizes and body weights. Animals were first anaesthetized in 0.03% tricaine methane sulfonate (MS-222, Sigma-Aldrich) in fish water and injected intraperitoneally (volume: 2 μl) with saline, muscarine (50 μΜ; 525 ng/g body weight) or/and methoctramine (40 μM; 2230 ng/g body weight; Fig. 3A). Treated animals were placed in the swim tunnel (5 L; Loligo systems, Denmark) to recover and acclimated at a low water flow speed (4.5 cm/sec) for 7 min. After, fish were given the U_crit_ test, subjecting the animals to time intervals of a certain flow velocity (increments of 4.5 cm/sec in 5 min steps) until the fish could not swim against the water current (fatigued; Fig. S4B). Fatigue was determined when fish stopped swimming and was forced against the rear net of the tunnel for more than 5 sec. Critical speed was then calculated using the following equation^30^ (Eq. 1):

1 $${{\rm{U}}}_{{\rm{crit}}}={{\rm{U}}}_{{\rm{fatigue}}}+{[{\rm{U}}}_{{\rm{step}}}\times {({\rm{t}}}_{{\rm{fatigue}}}{/{\rm{t}}}_{{\rm{step}}})]$$

where (Eq. 1): U_fatigue_ = the highest flow velocity where fish swam the whole interval, U_step_ = velocity increment, t_fatigue_ = time elapsed at final velocity that fish swam in the last interval, t_step_ = time increment that is the duration of one interval. The critical speed was normalized to body length (BL) of the experimental animals and is given as BL/sec.

For the open field test, treated animals were placed in small dishes (diameter: 8 cm) and allowed to swim freely, while their swimming was recorded for 4 min. Analysis of 2 min swimming behavior was performed after optimization and implementation of wrMTrck, a freely available ImageJ plugin. The average velocity and maximum velocity was normalized to body length (BL) of the experimental animals and is given as BL/sec.

### Statistical analyses

The significance of differences between the means in experimental groups and conditions was analyzed using the *One-way* ANOVA followed by *post hoc* Tukey test and the two-tailed Student’s *t*-test (paired or unpaired), using Prism (GraphPad Software Inc.) Significance levels indicated in all figures as follows: *p < 0.05, **p < 0.01, ***p < 0.001, ****p < 0.0001. Data presented here are given as mean ± SD, and as box (mean) and whiskers (min to max). Finally, the n values reflect the final number of validated animals per group or the number of cells that have evaluated.

## Electronic supplementary material

Supplementary information
