# Supplementary material for: Spinal cholinergic interneurons differentially control motoneuron excitability and alter the locomotor network operational range
Source: Sci Rep. 2018 Jan 31;8:1988. doi: 10.1038/s41598-018-20493-z (PMC5792632; doi:10.1038/s41598-018-20493-z)
Supplement: Supplementary file 1 — Supplementary information [file 41598_2018_20493_MOESM1_ESM.pdf]

# Spinal cholinergic interneurons differentially control motoneuron excitability and alter the locomotor network operational range

Maria Bertuzzi & Konstantinos Ampatzis

Department of Neuroscience, Karolinska Institutet, 171 77 Stockholm, Sweden

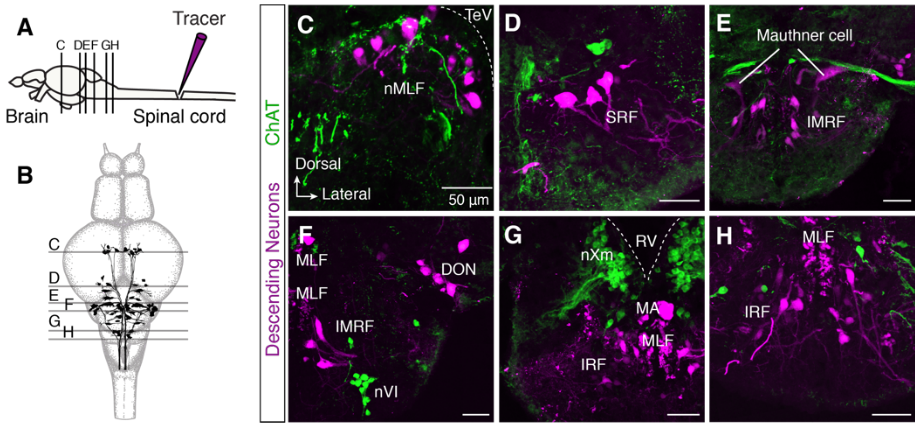


**Figure S1. Brain neurons descending to spinal cord are not cholinergic.** (**A-B**) Injection of methylrhodamine dextran in the spinal cord retrogradely labels all the descending supra-spinal neurons (N = 8 zebrafish brains). Schematic representation of the distribution of adult zebrafish brain descending neurons with the level of sections that correspond to the following images in C-H. (**C-H**) Confocal images show that none of the descending labeled neuron is ChAT$^{+}$ in all studied brain areas. DON, descending octaval nucleus; IMRF, intermediate reticular formation; IRF, inferior reticular formation; MA, Mauthner axon; MLF, medial longitudinal fascicle; nMLF, nucleus of the medial longitudinal fascicle; nVI, abducens nucleus; nXm, vagal motor nucleus; RV, rhombencephalic ventricle; SRF, superior reticular formation; TeV, tectal ventricle.

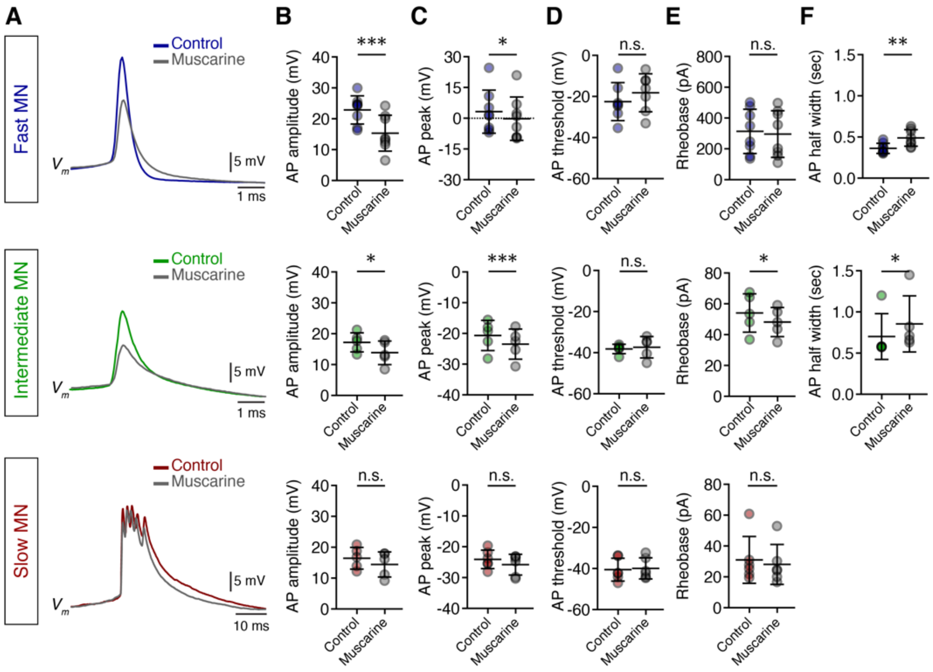


**Figure S2. Changes in MN electrical properties after the bath application of muscarine.** (**A**) Superimposed representative examples of the first action potential (AP) in fast and intermediate motoneurons and first burst of action potentials (APs) of slow motoneurons before (colored traces) and after muscarine (gray traces). (**B**) The AP amplitude was significantly reduced by muscarine in intermediate ($t = 4.33$, $p = 0.0123$, $n = 5$) and fast motoneurons ($t = 5.66$, $p = 0.0008$, $n = 8$). (**C**) Following the reduction of AP amplitude also the AP peak was found to be hyperpolarized (Fast MNs: $t = 2.44$, $p = 0.044$, $n = 8$; Intermediate MNs: $t = 14.64$, $p = 0.0001$). (**D**) Muscarine application was found not to affect the AP threshold. (**E**) Rheobase, the minimum current injection that generates an AP, was hyperpolarized in intermediate motoneurons ($t = 4.24$, $p = 0.013$, $n = 5$). (**F**) The half-width duration of the AP was significantly increased by muscarine in motoneurons firing single AP's (Intermediate MNs: $t = 3.79$, $p = 0.019$, $n = 5$; Fast MNs: $t = 4.9$, $p = 0.0017$, $n = 8$). Student's paired t test. Data are presented as mean ± SD; *$p < 0.05$; **$p < 0.01$; ***$p < 0.001$; n.s., non-significant.

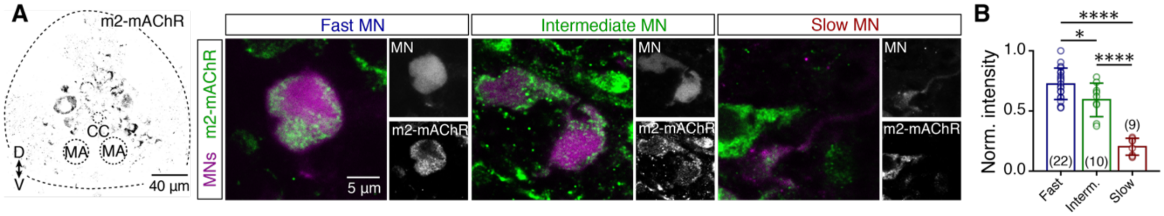


**Figure S3. m2-mAChRs in different motoneurons.** (**A-B**) Distribution pattern of m2-mAChRs in relation to different motoneuron types. A combination of retrograde labeling of different motoneuron pools with immunostaining for m2-mAChRs reveals that the fast motoneurons exhibit a vast number of m2 receptors compared to other motoneuron types (intermediate and slow). Slow motoneurons were found to be weakly labeled. Data are presented as mean ± SD; **p* < 0.05; *****p* < 0.0001.

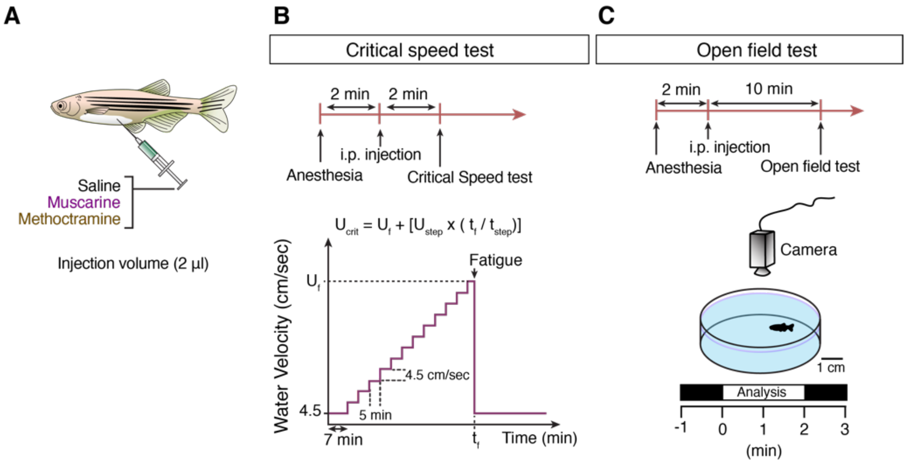


**Figure S4. Experimental design to investigate the effect of mAChRs during *in vivo* locomotion.** (**A**) Intraperitoneal administration of saline, muscarine and/or methoctramine in anesthetized adult zebrafish before the *in vivo* tests. (**B**) The critical speed test protocol. (**C**) Protocol for *in vivo* monitoring of adult zebrafish locomotor behavior.
